# OBJECTIVELY MEASURED PHYSICAL ACTIVITY PATTERNS OVER 24-HOURS BY APOE-Ε4 CARRIER STATUS

**DOI:** 10.1093/geroni/igac059.1305

**Published:** 2022-12-20

**Authors:** Amal Wanigatunga, Francesca Marino, Fangyu Liu, Eleanor Simonsick, Qu Tian, Jennifer Deal, Susan Resnick, Jennifer Schrack

**Affiliations:** Johns Hopkins Bloomberg School of Public Health, Baltimore, Maryland, United States; Johns Hopkins Bloomberg School of Public Health, Washington, District of Columbia, United States; Johns Hopkins Bloomberg School of Public Health, Baltimore, Maryland, United States; National Institute on Aging, Baltimore, Maryland, United States; National Institutes on Aging, Baltimore, Maryland, United States; Johns Hopkins University, Baltimore, Maryland, United States; National Institute on Aging, Baltimore, Maryland, United States; Johns Hopkins Bloomberg School of Public Health, Baltimore, Maryland, United States

## Abstract

The Apolipoprotein-ε4 (APOE-ε4) genotype is associated with earlier onset and greater risk of β-amyloid deposition, but whether APOE-ε4 status is associated with physical activity amount or fragmentation remains unclear. In 563 BLSA participants aged 55-97 years with APOE-ε4 and ≥3 days of wrist accelerometry data, multivariable linear regression models were fitted to estimate differences in activity amount (daily or by 6-hour time blocks) or fragmentation by APOE-ε4 status and to test whether age modifies these effects. Overall, daily activity amount and fragmentation did not differ by APOE-ε4 status (p=0.28 and p=0.93, respectively). Yet, age significantly modified the APOE-ε4 effect. Per each additional year of age, APOE-ε4 carriers had less activity during early morning (12:00-6:00AM) than non-carriers (p=0.035). This association was non-significant among the oldest-old (p=0.697 at age 80 years, p=0.347 at age 90 years), suggesting possible decreases in the predictive power of APOE-ε4 with increasing age.

